# Supplementary material for: Association between oral microbiome and seven types of cancers in East Asian population: a two-sample Mendelian randomization analysis
Source: Front Mol Biosci. 2023 Nov 21;10:1327893. doi: 10.3389/fmolb.2023.1327893 (PMC10702768; doi:10.3389/fmolb.2023.1327893)
Supplement: Supplementary file 1 [file DataSheet1.ZIP › Supplementary Materials/MR plots for tongue/tongue═╝/Colorectal cancer/pheno.860_to_colorectal cancer_leave_one_out.pdf]

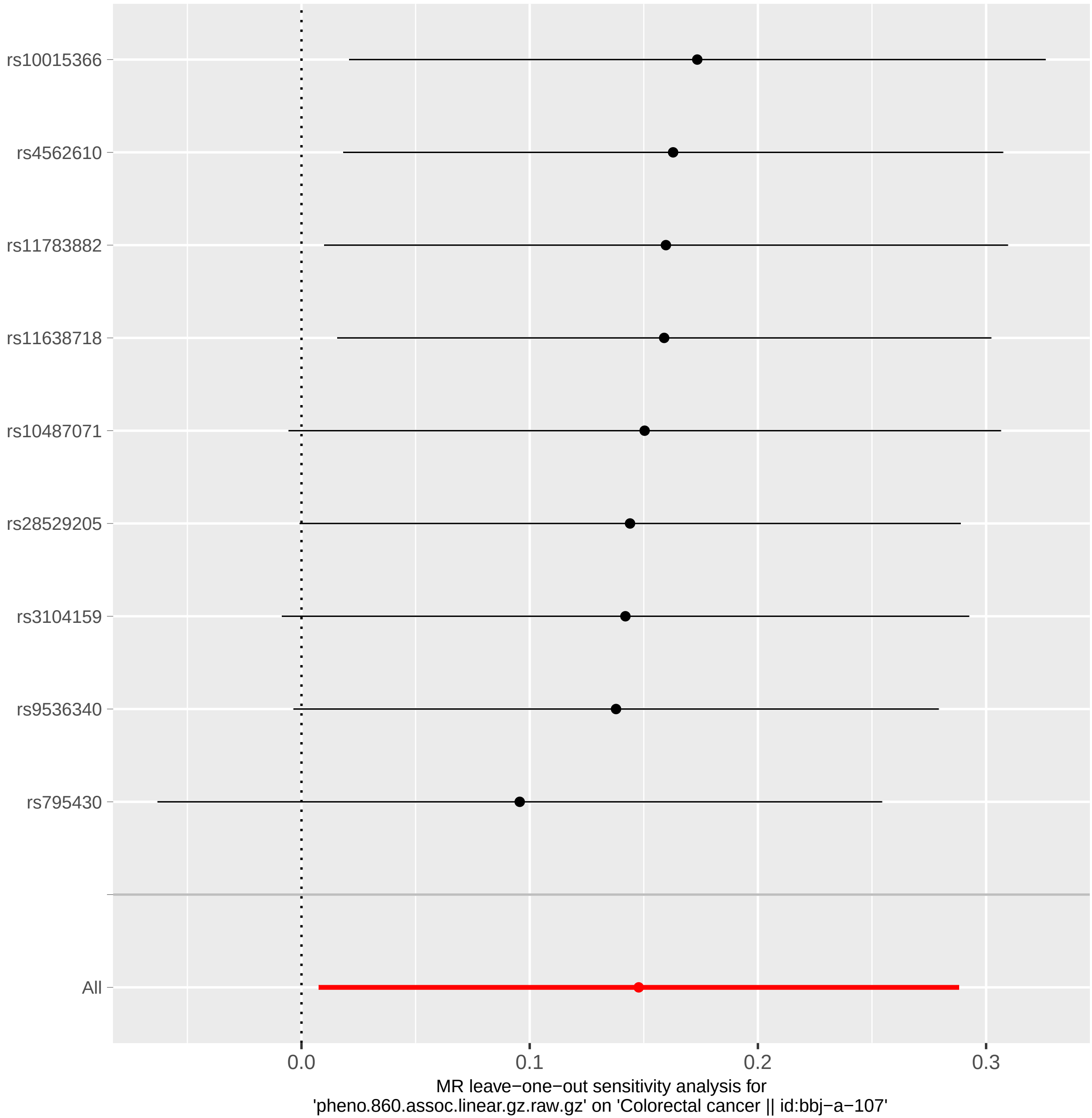
rs10015366
rs4562610
rs11783882
rs11638718
rs10487071
rs28529205
rs3104159
rs9536340
rs795430
All
0.0
0.1
0.2
0.3
MR leave−one−out sensitivity analysis for
'pheno.860.assoc.linear.gz.raw.gz' on 'Colorectal cancer || id:bbj−a−107'
